# Quality of YouTube videos on Chagas disease: compliance with clinical guidelines

**DOI:** 10.1038/s41598-026-50600-4

**Published:** 2026-05-01

**Authors:** Whesley Tanor Silva, Mauro Felippe Felix Mediano, Diêgo Mendes Xavier, Jéssica Stéfany Rocha, Lucas Frois Fernandes de Oliveira, Matheus Ribeiro Ávila, Rudson Santos da Silva, Murilo Xavier Oliveira, Laia Ventura Garcia, Mario Javier Olivera, Pedro Henrique Scheidt Figueiredo, Henrique Silveira Costa

**Affiliations:** 1 Postgraduate Course in Reabilitação e Desempenho Funcional, Universidade Federal dos Vales do Jequitinhonha e Mucuri (UFVJM), Diamantina, MG Brazil; 2 Oswaldo Cruz Foundation, Instituto Nacional de Infectologia Evandro Chagas, Avenida Brasil, 4365, Rio de Janeiro, RJ CEP 21040-360 Brazil; 3 Physical Therapy Department, Universidade Federal dos Vales do Jequitinhonha e Mucuri (UFVJM), Diamantina, MG Brazil; 4 Postgraduate Course in Infectologia e Medicina Tropical, Universidade Federal de Minas Gerais (UFMG), Belo Horizonte, MG Brazil; 5 Department of Anthropology, Philosophy and Social Work, Medical Anthropology Research Center (MARC), Universitat Rovira I Virgili, Barcelona, Spain; 6 Grupo de Parsitología, Instituto Nacional de Salud, Bogotá, Colombia

**Keywords:** Chagas disease, Science communication, Online health information, Health education, YouTube, Misinformation

## Abstract

Chagas disease remains a public health problem, and YouTube is used to access health information. This study aimed to assess whether YouTube videos on Chagas disease comply with scientific guidelines. This study analyzed videos identified using terms in English, Portuguese and Spanish. Two independent reviewers assessed eligible videos. Video quality was evaluated using a scale developed and pre-tested by three clinical experts. Comparisons between adequate and inadequate videos were performed using Mann–Whitney or Fisher’s exact tests, and associations between variables were assessed using Poisson regression. Among 158 videos screened, 96 were included. For definition, videos from healthcare and academic institutions/professionals showed higher prevalence ratio of adequate quality (1.86; 95% CI 1.14–3.02), as did treatment (3.69; 95% CI 1.67–8.15). Videos in English (0.57; 95% CI 0.34–0.97) and Spanish (0.31; 95% CI 0.16–0.61), and longer duration (0.998; 95% CI 0.995–0.999) were associated with lower quality for definition. Higher numbers of views, likes, and longer duration were associated with higher prevalence of adequate quality for etiology, while longer duration was associated with natural history and with lower prevalence of adequate quality for diagnosis. Most videos showed inadequate quality, highlighting the need for better guideline-based content on YouTube.

*Registration number*: 10.17605/OSF.IO/NXW8H

**Supplementary Information:**

The online version contains supplementary material available at 10.1038/s41598-026-50600-4.

## Introduction

Chagas disease is a major public health problem, primarily in Latin America, but also with an increasing prevalence in non-endemic regions such as the United States and Europe^1–4^. It is considered one of the most prevalent tropical neglected diseases according to the World Health Organization, affecting approximately 6 to 8 million people worldwide^5^. The infection is caused by the protozoan *Trypanosoma cruzi*, which triggers a series of immunological reactions in the host^6^. After infection, the individual enters the acute phase of the disease that lasts approximately 8–12 weeks, and subsequently progresses to the chronic phase^4^. In the chronic phase, patients may remain asymptomatic or develop clinical manifestations of the disease, which include cardiac, digestive, or mixed (both simultaneously) forms^7^.

Patients with chronic diseases are increasingly turning to internet-based resources to obtain health information^8^. This can contribute to the dissemination of health information and empower patients in their self-care; however, it can also expose them to inadequate information from unreliable sources^9,10^. For example, one previous study, focused on a different condition, showed that videos published on YouTube do not meet the guidelines recommendations^10^. In the case of Chagas disease, a previous study demonstrated that patients interacted through comments on several videos about their condition on YouTube and that the content of these comments revealed their limited understanding about the disease. However, the reliability of the information available on this platform remains unclear^11^. This is particularly concerning, as YouTube has more than 2 billion active users each month^12^, making this platform one of the most accessed sources of health information by the general population^13^.

Furthermore, Chagas disease often affects socially vulnerable patients in underdeveloped regions with limited resources. A theoretical model on the impact of Chagas disease on health-related quality of life indicated that individuals with this condition usually suffer from insufficient access to information. The lack of information begins at the time of diagnosis and persists throughout life, manifesting fears of transmission through social contact, concerns about prognosis, and limited understanding and fear of treatment^14^. Since patients often face difficulties in accessing reliable information from health institutions, they are turning to online platforms, particularly YouTube. In this setting, considering that misinformation can negatively affect quality of life, as well as patients’ physical and emotional well-being and health, it is essential to verify the quality of the information about Chagas disease available on this major platform. Therefore, the present study aimed to assess whether the information in YouTube videos complies with current guidelines for the diagnosis and treatment of Chagas disease.

## Methods

This cross-sectional study assessed the quality of information provided by the most viewed videos on the YouTube platform about Chagas disease. The present study followed the STROBE (Strengthening the Reporting of Observational Studies in Epidemiology) guidelines^15^ and was prospectively registered in the Open Science Framework. The YouTube searches were conducted in December 2022 (updated in February 2025). The use of videos stored on YouTube does not require approval from the Ethics Committee. However, the authors of the study guarantee the confidentiality of the authors of the included videos and state that the data will be used exclusively for this research (supplementary material, appendix 1).

### Searches, inclusion criteria, and recruitment

The electronic search was performed on the YouTube search engine using the descriptors “doença de Chagas”, “Chagas disease” and “La enfermedad de Chagas”. Reviewers were instructed to sign out of their Google accounts to prevent search history and data synchronization from influencing the search results. The researchers then used the filter available on the platform, selecting the “video” category and sorting by “relevance” to ensure that the first videos were the most related, reducing the impact of YouTube’s algorithm.

The selected videos containing information about Chagas disease were included. Duplicate videos, either partially or completely, and videos in languages other than English, Portuguese, or Spanish were excluded. Two reviewers evaluated all videos (DMX and WTS). Any disagreements were resolved by a third evaluator (HSC). The 50 most relevant videos (according to the platform’s filter) in each of the three languages (English, Portuguese, and Spanish) were selected, totaling 150 videos. This method of video selection is commonly used in the literature, as users rarely go beyond the first 50 search results^16^.

### Data extraction

The data collected about video characteristics included: (1) number of views; (2) number of likes; (3) video duration; (4) country of origin; (5) upload source; (6) language. The upload source was categorized as hospitals and academic institutions, health professionals, international organizations, government, or other sources (including personal channels, and independent content creators).

### Quality of videos

#### Development of the quality scale and pre-test

Three researchers held a videoconference to develop a scale identifying the items to be scored within each topic based on the established guidelines. After the development of the initial version, the scale underwent a pilot test to evaluate its applicability and the reliability of the scoring system. An initial evaluation was conducted with 12 videos (4 in each predetermined language). Based on the needs identified in the pilot study, the experts adapted the scale until the final version was developed. The main adaptations were related to the scoring system. The versions of the scale are available in the supplementary material (appendices 2 and 3). The final version of the scale has five key topics to assess compliance with the major guidelines: (1) definition, (2) etiology, (3) natural history of the disease, (4) diagnosis, and (5) treatment^17–21^. These topics were selected as they cover essential topics that support patient self-management of the Chagas disease. The scale scored videos based on their compliance with scientific guidelines. Each topic was scored from 1 (basic mention of key concepts) to 3 (detailed description of all guideline elements), except for the definition topic, which was assessed as a dichotomous variable (adequate/inadequate) due to the difficulty of objectively establishing intermediate points of reference for this topic.

#### Quality assessment and classification through guideline compliance

Two independent reviewers assessed the included videos using the developed scale. Discrepancies were resolved through consensus meetings; when consensus could not be reached, a third reviewer made the final determination. For each item, scores ≥ 2 were classified as demonstrating adequate quality according to the guidelines, while scores of 1 were considered of inadequate quality. This threshold was based on the 60% benchmark commonly used in scientific literature for bias assessment^22^. The items analyzed, expected answers, and classification/score are described in appendix 3 of supplementary material.

### Data analysis

Data analyzes were performed using the SPSS statistical package, version 25.0 (Inc., USA) and R software (version 3.6.3) using the packages: readxl and stats for Fisher’s exact test)]. Data normality was verified by the Kolmogorov–Smirnov test, and data were presented as the mean and standard deviation for variables with normal distribution and as median and interquartile range for variables with non-normal distribution. Categorical variables were presented in absolute numbers and percentages.

To facilitate data analysis, the variable country of origin was categorized as endemic countries (Latin America) and non-endemic countries (North America, Europe, and others). The variable upload source was grouped into healthcare and academic institutions/professionals, governmental/intergovernmental organizations, and others (e.g. independent educational channels, lay users, or personal channels producing independent content). Finally, the continuous variables were scaled to facilitate interpretation (duration: per 5 min, views: per 1000, and likes: per 100 units). Differences in video characteristics between the adequate and inadequate quality groups were compared using the Mann–Whitney test for continuous variables. Pearson’s chi-square test was used for comparisons between categorical variables, and Fisher’s exact test was applied when expected cell counts were less than 5 (e.g., comparing the proportion of adequate vs. inadequate videos among those in Portuguese).

The associations between video characteristics with video quality were examined using Poisson models with robust variance, using each topic (definition, etiology, natural history of the disease, diagnosis and treatment) score—dichotomized into adequate and inadequate quality—as the dependent variable. Independent variables with a significance value ≤ 0.10 in the univariate models were included in the multivariate model, which maintained only those variables with a significance value lower than 0.05 using a stepwise backward approach. A significance level of 0.05 was adopted for the analysis.

## Results

The pre-test of the 5-topic scale showed high agreement, ranging from 83.3% to 100%, with Cohen’s kappa coefficients ranging from 0.62 to 1.00. The agreement analysis is presented in Supplementary Table 2.

**Fig. 1:**
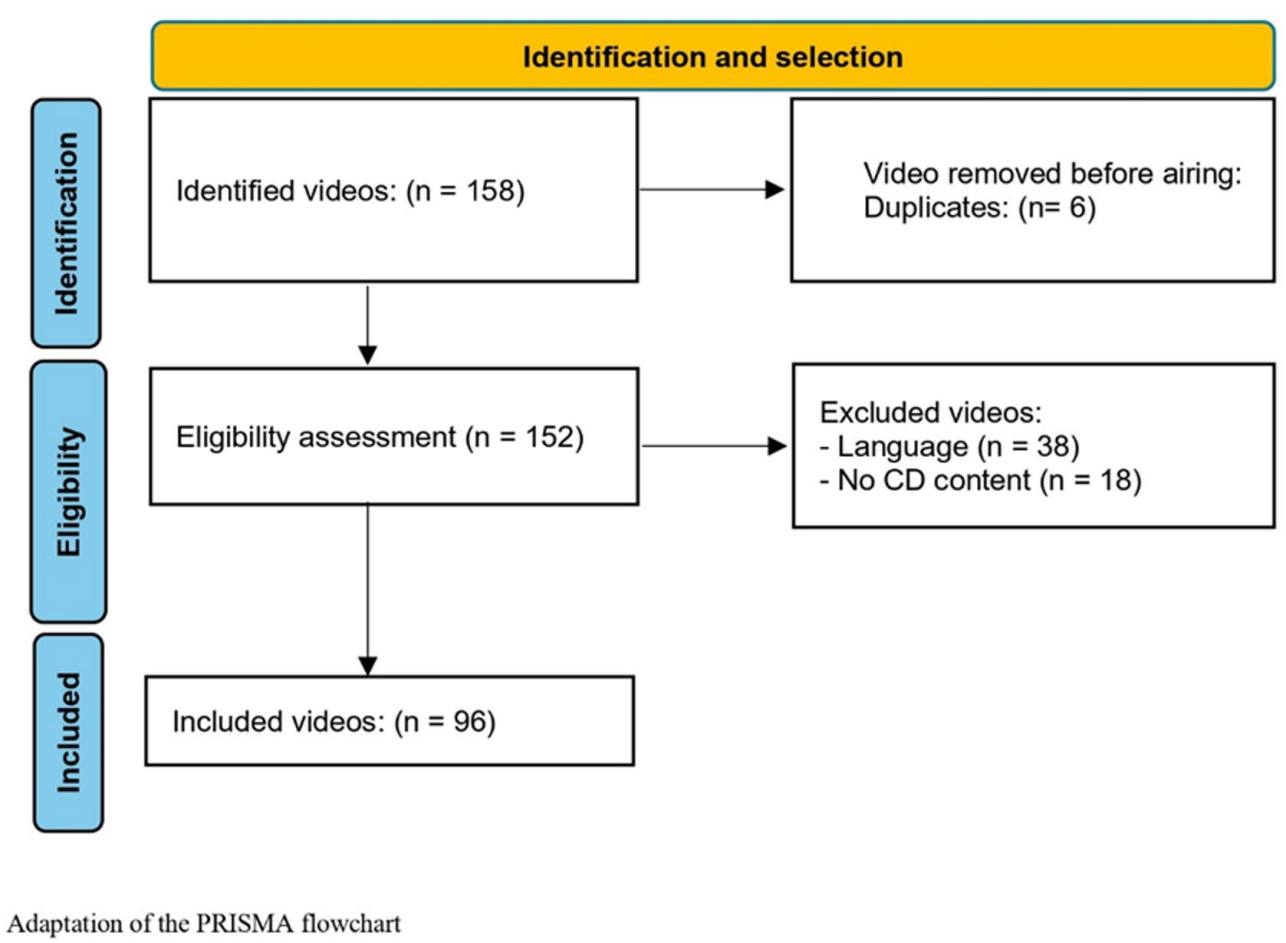
Flowchart of videos in the study.

Among the 158 videos selected, six were duplicates, 38 were not in English, Portuguese, or Spanish, and 18 had no information about Chagas disease. The remaining 96 videos met the inclusion criteria and were included in the study. Figure 1 depicts the flowchart of videos included in the study.

### Characteristics of the included videos

Information regarding the definition of the disease was presented in all videos, while etiology was addressed in 96.0%, followed by treatment in 64.4%, natural history of disease in 57.3%, and diagnosis in 30.2%. The videos were uploaded between the years 2012 and 2024.

Overall, the videos had a median of 8,425.5 views, 147.5 likes, and a duration of 4.49 min. Most videos originated from Brazil (49.0%) and were primarily uploaded from other sources (41.7%), followed by hospital/academic institutions (21.9%) and international organizations (17.7%). The most common video language was Portuguese (47.9%). The detailed characteristics of the 96 included videos are presented in Supplementary Table 3.

**Fig. 2:**
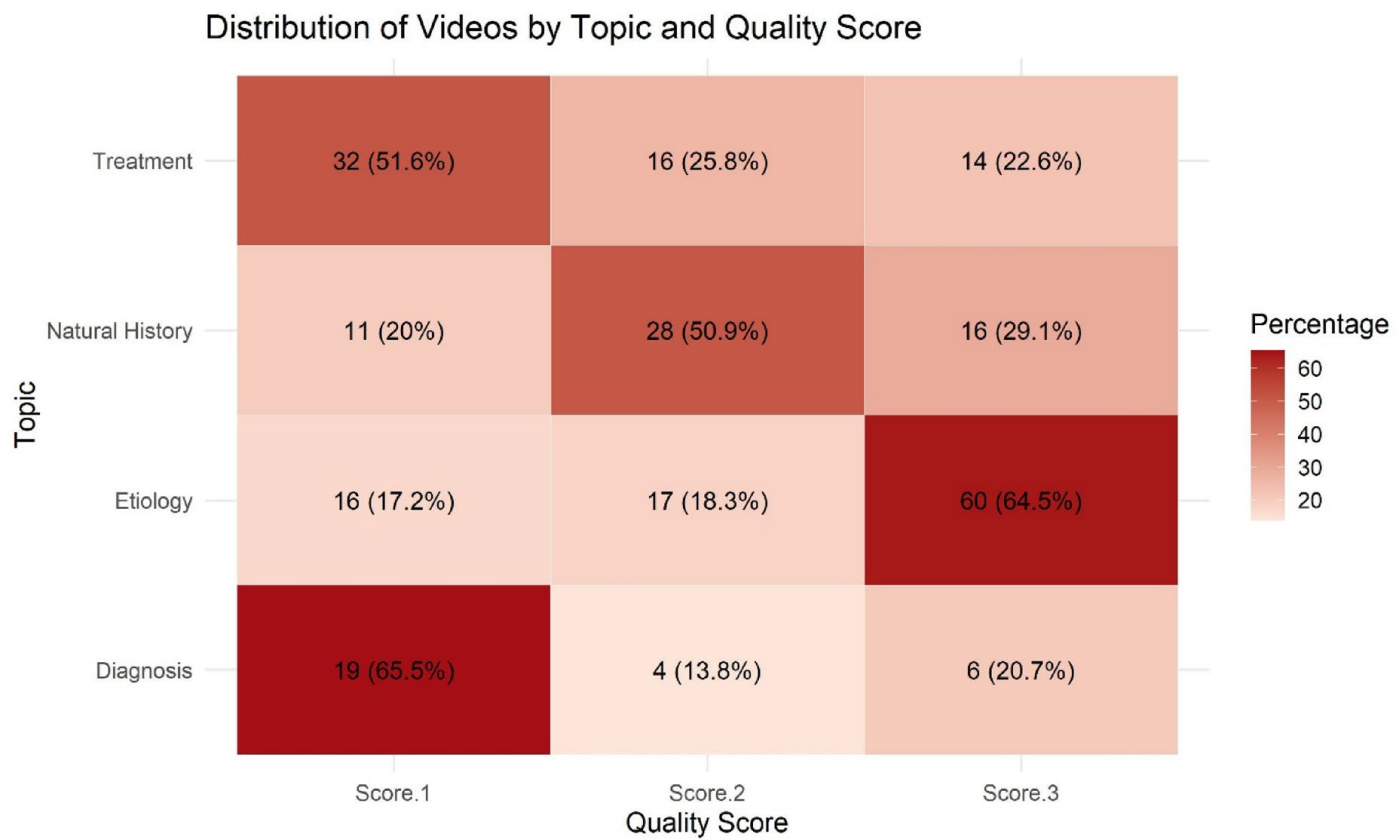
Relative distribution of YouTube video quality scores across topics related to Chagas disease.

Table 1 presents the distribution of videos according to quality scores (1 to 3) for each topic, as well as the median and 25–75% interquartile range of scores. The quality of information was considered adequate in 82.8% of the videos addressing etiology, 70.0% covering the natural history of the disease, and 48.4% of those about treatment. Figure 2 shows the relative distribution of quality scores for YouTube videos across the investigated topics (except for definition, that was assessed as a dichotomous variable).

**Table 1:** Characteristics of the included videos (n = 96).

Variable	Values
Views (median, 25–75%IQR)	8425.50 (1036.25–42,499.25)
Likes (median, 25–75%IQR)	147.50 (17.50–862.25)
Duration in min (median, 25–75%IQR)	4.49 (2.69–20.33)
Country of origin (n, %)	
Brazil	47 (49.00%)
USA	11 (11.50%)
Chile	1 (1.00%)
Argentina	7 (8.61%)
El Salvador	2 (2.10%)
Spain	9 (9.40%)
Peru	1 (1.00%)
Switzerland	4 (4.50%)
Colombia	1 (1.00%)
Canada	2 (2.10%)
UK	1 (1.00%)
Mexico	1 (1.00%)
Australia	1 (1.00%)
Denmark	1 (1.00%)
Upload source (n, %)	
Healthcare and academic institutions/professionals	27 (28.1%)
Governmental/intergovernmental organizations	29 (30.2%)
Others	40 (41.7%)
Language	
Portuguese	46 (47.9%)
Spanish	32 (33.3%)
English	18 (18.8%)

Data are shown as median and 25–75% interquartile range or absolute frequency and percentage. USA: United States of America; UK: United Kingdom.

Video characteristics according to adequate and inadequate quality are presented in Table 2. The duration of the videos was longer in videos with adequate quality on all items, except natural history of the disease. Regarding language, Portuguese videos were more frequently adequate in the definition topic, whereas Spanish videos were more often inadequate in this same topic. For country of origin, videos from endemic regions were more frequently adequate for etiology. Regarding the upload source, videos produced by healthcare and academic institutions/professionals were more frequently adequate for treatment, while those from other sources were more frequently inadequate for this same topic.

**Table 2:** Compliance between YouTube video content and current guidelines for Chagas disease management (5-Topic Scale).

Topics	Number of videos that covered the topic/scoring frequency	Median score
Definition	96 (100%)	
Inadequate	50 (52.10%)	
Adequate	46 (47.90%)	
Etiology (maximum: 3)	93 (96.00%)	3.00 (2–3)
1 point	16 (17.20%)	
2 points	17 (18.30%)	
3 points	60 (64.50%)	
Natural history of the disease (maximum: 3)	55 (57.30%)	2.00 (2–3)
1 point	11 (20.00%)	
2 points	28 (50.90%)	
3 points	16 (29.10%)	
Diagnosis (maximum: 3)	29 (30.20%)	1.00 (1–2)
1 point	19 (65.50%)	
2 points	4 (13.80%)	
3 points	6 (20.70%)	
Treatment (maximum: 3)	62 (64.60%)	1.00 (1–2)
1 point	32 (51.60%)	
2 points	16 (25.80%)	
3 points	14 (22.60%)	

Data are shown as median and 25–75% interquartile range or absolute frequency and percentage.

The univariate analyses evaluating the association between video characteristics and video quality (adequate vs inadequate) are presented in Supplementary Table 4. For definition, there was an association between longer duration (PR = 0.998, 95% CI 0.996 to 0.999, *p*-value = 0.04) and Spanish videos with lower prevalence of adequate quality (PR = 0.33, 95% CI 0.16 to 0.67, *p*-value = 0.02). Conversely, a higher number of views (PR = 1.001, 95% CI 1.000 to 1.002, *p*-value = 0.006), number of likes (PR = 1.005, 95% CI 1.001 to 1.007, *p*-value = 0.003), as well as longer duration (PR = 1.000, 95% CI 1.000 to 1.001, *p*-value < 0.001), were associated with a higher prevalence of adequate quality in etiology topic. For natural history of the disease and diagnosis, only the duration of videos was associated with video quality, being linked to higher prevalence of adequate quality in natural history of the disease (PR = 1.001, 95% CI 1.000 to 1.001, *p*-value = 0.001) and to lower prevalence of adequate quality in diagnosis (PR = 0.998, 95% CI 0.996 to 0.999, *p*-value = 0.03). Finally, in the treatment topic, duration (PR = 1.001, 95% CI 1.000 to 1.001, *p*-value < 0.001), English language (PR = 2.12, 95% CI 1.18 to 3.81, p = 0.01), and uploads source from healthcare and academic institutions/professionals (PR = 3.22, 95% CI 1.57 to 6.59, *p*-value = 0.001) were associated with a higher prevalence of adequate video quality (Table 3).

**Table 3:** Comparison of video characteristics with adequate and inadequate quality.

Variable	Adequate	Inadequate	*p*-value
Definition (n = 96)			
Views	8226.5 (1052.8–36,598.8)	12,528.5 (715.8–58,796.3)	0.54
Likes	154.0 (23.5–1125.0)	139.5 (12.8–854.8)	0.58
Duration (min)	7.56 (3.51–35.38)	4.18 (2.20–11.41)	0.02
Language			
Portuguese	30 (65.2)	16 (32.0)	0.001
Spanish	7 (15.2)	25 (50.0)	< 0.001
English	9 (19.6)	9 (18.0)	0.84
Country of origin			
Endemic country	35 (76.1)	30 (60.0)	0.92
Non-endemic country	11 (23.9)	20 (40.0)	0.92
Upload source			
Healthcare and academic institutions/professionals	16 (34.8)	11 (22.0)	0.16
Governmental/intergovernmental organizations	15 (32.6)	14 (28.0)	0.62
Others	15 (32.6)	25 (50.0)	0.08
Etiology (n = 93)			
Views	10,187.0 (1044.5—45,687.5)	3893.5 (473.8—20,773.8)	0.14
Likes	153.0 (15.0—874.5)	35.0 (24.0—534.8)	0.32
Duration (min)	5.34 (3.22—27.27)	3.17 (1.42—7.39)	0.01
Language			
Portuguese	36(46.8)	9 (56.3)	0.48
Spanish	26 (33.8)	4 (25.0)	0.49
English	15 (19.5)	3 (18.8)	0.94
Country of origin			
Endemic country	54 (70.1)	10 (62.5)	0.05
Non-endemic country	23 (29.9)	6 (37.5)	0.54
Upload source			
Healthcare and academic institutions/professionals	24 (31.2)	3 (18.8)	0.38
Governmental/intergovernmental organizations	21 (27.3)	6 (37.5)	0.41
Others	32 (41.6)	7 (43.8)	0.87
Natural history of disease (n = 55)			
Views	14,255.0 (1185.0—48,351.0)	4068.5 (2250.0—25,942.5)	0.42
Likes	211.0 (38.0—687.0)	46.5 (14.5—977.0)	0.23
Duration (min)	7.12 (3.51—33.00)	4.97 (2.64—38.73)	0.63
Language			
Portuguese	18 (41.9)	5 (41.7)	0.99
Spanish	17 (39.5)	5 (41.7)	0.89
English	8 (18.6)	2 (16.7)	0.87
Country of origin			
Endemic country	29 (67.4)	8 (66.7)	1.00
Non-endemic country	14 (32.6)	4 (33.3)	1.00
Upload source			
Healthcare and academic institutions/professionals	15 (34.9)	3 (25.0)	0.73
Governmental/intergovernmental organizations	12 (27.9)	5 (41.7)	0.36
Others	15 (34.9)	3 (25.0)	0.73
Diagnosis (n = 29)			
Views	7126.5 (1668.8–41,566.0)	8451.0 (866.0–68,515.0)	0.81
Likes	113.5 (48.5–2025.0)	190.0 (8.0–754.0)	0.90
Duration (min)	45.43 (31.60–59.35)	8.00 (4.00–27.50)	0.01
Language			
Portuguese	5 (50.0)	8(42.1)	0.68
Spanish	5 (50.0)	6 (31.6)	0.33
English	0 (0.00)	5 (26.3)	0.34
Country of origin			
Endemic country	9 (90.0)	11 (57.9)	0.11
Non-endemic country	1 (10.0)	8 (42.1)	0.11
Upload source			
Healthcare and academic institutions/professionals	5 (50.0)	8 (42.1)	0.68
Governmental/intergovernmental organizations	3 (30.0)	5 (26.3)	1.00
Others	2 (20)	6 (31.6)	0.67
Treatment (n = 62)			
Views	5482.5 (819.3–23,875.0)	4506.5 (1112.8–52,646.3)	0.43
Likes	120.0 (7.5–556.5)	103.5 (20.3–848.0)	0.65
Duration (min)	11.99 (4.29–51.09)	4.51 (2.55–19.24)	0.04
Language			
Portuguese	11 (36.7)	19 (59.4)	0.07
Spanish	12 (40.0)	11 (34.4)	0.64
English	7 (23.3)	2 (6.3)	0.07
Country of origin			
Endemic country	19 (63.3)	25 (78.1)	0.20
Non-endemic country	11 (36.7)	7 (21.9)	0.20
Upload source			
Healthcare and academic institutions/professionals	16 (53.3)	3 (9.4)	< 0.001
Governmental/intergovernmental organizations	8 (26.7)	12 (37.7)	0.36
Others	6 (20.0)	17 (53.1)	0.007

Data are shown as median and 25–75% interquartile range or absolute frequency and percentage.

The variables that remained independently associated in the multivariate models for each topic are depicted in Table 4. For definition, videos from healthcare and academic institutions/professionals (PR = 1.86, 95% CI 1.14 to 3.02, p = 0.01) were associated with higher prevalence of adequate video quality, while English (PR = 0.57, 95% CI 0.34 to 0.97, p = 0.03), Spanish (PR = 0.31, 95% CI 0.16 to 0.61, p = 0.01), and longer duration (PR = 0.998, 95% CI 0.995 to 0.999, p = 0.02) were associated with a lower prevalence. For etiology, the number of views per 1,000 units (PR = 1.000, 95% CI 1.000 to 1.001, p = 0.02), likes per 100 units (PR = 1.003, 95% CI 1.0001 to 1.006, p = 0.03), and longer duration per 5 min (PR = 1.000, 95% CI 1.000 to 1.001, p < 0.001) were associated with higher prevalence of adequate videos. For natural history of disease and diagnosis, only duration per 5 min remained associated with higher prevalence of adequate quality (PR = 1.000, 95% CI 1.000 to 1.001, p = 0.001; and PR = 0.998, 95% CI 0.996 to 0.999, p = 0.03, respectively). Finally, for treatment, videos uploaded by healthcare and academic institutions/professionals (PR = 3.69, 95% CI 1.67 to 8.15, p = 0.002) and those with longer duration (PR = 1.001, 95% CI 1.001 to 1.002, p < 0.001) were significantly associated with higher prevalence of adequate quality. Final adjusted models are presented in Table 4, whereas the complete multivariable models before final model reduction are provided in Supplementary Table 5.

**Table 4:** Final multivariable model analyzing factors associated with video information quality.

Topic	Variable	Category	PR	95% CI	*p*-value
Definition	Upload source	Others	Reference		
		Governmental/intergovernmental organizations	1.47	0.92 to 2.35	0.11
		Healthcare and academic institutions/professionals	1.86	1.14 to 3.02	0.01
	Language	Portuguese	Reference		
		English	0.57	0.34 to 0.97	0.03
		Spanish	0.31	0.16 to 0.61	0.01
	Duration (per 5 min)		0.998	0.995 to 0.999	0.02
Etiology	Views (per 1000 units)		1.000	1.000 to 1.001	0.02
	Likes (per 100 units)		1.003	1.001 to 1.006	0.03
	Duration (per 5 min)		1.000	1.000 to 1.001	< 0.001
Natural history of disease	Duration (per 5 min)		1.000	1.000 to 1.001	0.001
Diagnosis	Duration (per 5 min)		0.998	0.996 to 0.999	0.03
Treatment	Upload source	Others	Reference		
		Governmental/intergovernmental organizations	1.75	0.68 to 4.47	0.24
		Healthcare and academic institutions/professionals	3.69	1.67 to 8.15	0.001
	Duration (per 5 min)		1.001	1.001 to 1.002	< 0.001

PR for Views is expressed per 1000 views; for Likes per 100 likes; for Duration per 5 min. Abbreviations: CI, confidence interval; PR, prevalence ratio; ref., reference category; NHD, natural history of disease.

## Discussion

To the best of our knowledge, this is the first study to evaluate the compliance of YouTube videos about Chagas disease with scientific guidelines. Our findings reveal significant variability in video quality across the topics investigated. Overall, while basic aspects such as definition and etiology were frequently addressed, other important areas—including natural history of disease, diagnosis, and treatment—were less consistently covered. The quality of information also varied, being adequate in most videos addressing etiology and natural history, but considerably lower for the other topics. Regarding country of origin, Brazil contributed to the highest number of videos, followed by the United States and Spain, likely reflecting Brazil’s endemicity and migration patterns to the United States and Spain.

In the definition topic, videos from healthcare and academic institutions/professionals were associated with higher quality, whereas being in Spanish or English was associated with inadequate quality. Regarding etiology, only duration, views, and likes were associated with video quality, with a higher probability of adequate quality in longer videos. Similarly, only video duration was associated with quality in the topics of natural history of the disease and diagnosis; however, in diagnosis, longer videos were more likely to present inadequate information. Finally, in the treatment topic, longer videos were also more likely to have adequate information, as well as those from healthcare and academic institutions/professionals, which were strongly associated with adequate quality.

Overall, the most-viewed YouTube videos about Chagas disease demonstrated inadequate compliance with clinical guidelines across most topics, with particularly critical gaps in diagnostic and treatment information. Previous studies in other populations have also found similar findings^16,23^. For instance, Osman et al.^24^, in a systematic review of studies evaluating information from the YouTube platform, highlighted that the health-related information is often unreliable, and metrics such as views and likes are not associated with high-quality content. This also occurred in our study, as there were no differences between the number of likes and views and the quality of the information, with only a slight association in the topic of the natural history of the disease. This suggests that metrics related to views and interactions with videos do not guarantee adequate quality^24^. In fact, videos containing inadequate information may be more attractive and widely viewed than those of good quality, as studies have reported an inverse relationship between these metrics and quality^25,26^.

Conversely, regarding video duration, there was an association between adequate quality and longer video durations for the topics of etiology, natural history of the disease, and treatment, suggesting that adequate coverage of guideline topics may require longer videos. This pattern is particularly pronounced for treatment topics, likely due to their inherent complexity, including stage-specific treatment approaches^17^. Consequently, videos addressing these items comprehensively tend to be longer, while shorter videos often lack critical information^27^ a finding consistent with our results, which show high proportions of inadequate videos for diagnosis (65.5%) and treatment (51.6%), with videos of acceptable quality on treatment being statistically more frequent in healthcare and academic institutions or among professionals. However, it should be emphasized that duration cannot always be considered a marker of quality, given that for other topics, such as definition and diagnosis, longer duration was associated with poorer quality. Longer videos may contain more adequate information specifically due to having more time to present it, but the same could happen to inadequate information, as previously mentioned by Li et al.^28^. This concern is more pronounced for the diagnosis topic than for the definition topic because, for diagnosis, video duration was the only variable associated with quality, demonstrating the scarcity of videos with adequate quality about this topic, as evidenced by our findings that diagnosis is both the least discussed topic and the one with the largest amount of inadequate information.

Importantly, the combination of low coverage (diagnosis: 30.2%; treatment: 64.6%) and inadequate quality (diagnosis: 65.5%; treatment: 51.6%) in these videos raises significant concerns. Given that these resources are directed for patients with Chagas disease, such deficiencies contradict established evidence showing that diagnostic uncertainty and treatment-related fears significantly impair quality of life in this population^29,30^. This is worrying and becomes an even more serious problem when considering data from the systematic review by Madathil et al.^8^, which shows YouTube has been used to disseminate non-scientific treatments and/or those that are still not approved by the bodies in each country. Furthermore, there is a high frequency of misinformation about topics not specifically related with Chagas disease such as vaccines, drugs, and medical treatments^31^, which can significantly affect patients with Chagas disease. Previous studies suggest that misinformation in health can have effects in increasing negative emotions, paranoid thinking, and emotional distress^32,33^. Thus, misinformation should be taken into consideration as a factor of poor health-related quality of life, as well as emotional impacts on these patients, who have high levels of anxiety and depression^34,35^.

Additionally, videos from trusted sources such as healthcare and academic institutions/professionals were more likely to demonstrate higher quality in the treatment topic compared to those from other sources. One possible explanation for this finding is that hospitals and academics typically have greater access to up-to-date scientific information and patient care guidelines. However, it must be considered that these institutions often use scientific and technical languages, which may be difficult for the general population to understand^36–38^. An additional noteworthy finding was that videos in Spanish and English were less likely to achieve adequate quality on the topic of definition. This may be attributed to the fact that Portuguese, used as the reference language in the analysis, is the dominant language in academia and scientific dissemination on Chagas disease, due to the large concentration of researchers on this condition in Brazil^39^. Other contributing factors may include the limited availability of high-quality scientific content in Spanish and may also reflect the smaller number of institutional or academic channels producing videos in Spanish. These findings underscore the need for targeted efforts to improve the accessibility and quality of information about Chagas disease for Spanish-speaking patients, especially considering that most endemic countries are Spanish-speaking.

This study also demonstrated that most of the YouTube videos of Chagas disease are from Brazil. This finding may be partly explained by the extensive network of Brazilian researchers dedicated to the management of the condition^40^. Although Chagas disease is highly endemic in Latin America^4^, the second country with the highest number of informative videos about the disease is the United States, followed by Spain, which is explained by the intense recent migratory flow of patients^4^. Although this study did not assess the full set of videos available on YouTube, focusing instead on the 50 videos ranked as most relevant by the platform, this finding highlights the need for Latin American countries to invest in disseminating information about Chagas disease on YouTube, a task that can be undertaken by educational and governmental institutions, among others^41^. This is particularly relevant in the case of countries such as Bolivia, which has a high incidence of Chagas disease, yet no educational videos on Chagas disease from Bolivia were found in our sample^4^.

These findings are even more concerning when considering that Latin American and Spanish videos are more likely to contain inadequate quality information in the topics of ‘natural history of disease’ and ‘definition.’ In other words, in addition to the limited number of videos from Latin American countries, with the exception of Brazil, there is an association between inadequate quality videos in the topic of ‘natural history of disease.’ Furthermore, Spanish videos, which are among the most frequent, are associated with inadequate quality in the topic of ‘definition.’ This low number of videos per country in Latin America may be explained by the predominance of the Spanish language in the region, which allows videos from one country to be accessed and used by populations in another. However, we emphasize the need for greater efforts to produce adequate quality videos in Latin American countries and Spain.

Furthermore, scientific communication on Chagas disease and health in general can be more effective by adopting a set of strategies. Research funding agencies should not only support but actively encourage researchers to engage in scientific communication, as financial incentives can serve as a key motivator to recognize and reward this additional responsibility^42^. It is also essential to establish training programs that provide researchers with the skills to communicate complex findings in accessible ways, which has been shown to enhance public understanding and engagement^43^. These strategies should involve students in health courses, providing dedicated scholarships or grants for undergraduate students to focus on scientific communication, which can help generate a new generation of researchers and clinicians committed to scientific communication.

Finally, we believe there is an urgent need for incentives to promote the production of high-quality content on Chagas disease for health education on YouTube. For this reason, professionals who work with information and communication in health need to be trained on the condition, that can be provided by health professionals or researchers with expertise in this condition^36^. Furthermore, the producers of content on Chagas disease must always translate the technical language into popular language. In this sense, the use of qualitative research with patients with Chagas disease can be a valuable tool to understand their desires and the best communication strategies^44^, and this will make it easier to circulate appropriate information^36^.

This study has strengths and limitations. We suggest that future studies adopt a scoring system that attributes points for content consistent with guidelines, but also deducts points when information is incorrect or misleading. We also highlight that video relevance rankings are dynamic, meaning these findings correspond to the time of the search, which is a limitation of the study’s cross-sectional design, as the results may vary over time. As a strength, this study adopted a consistent methodological framework, similar to that used in systematic literature reviews, to reduce potential biases as much as possible. An additional limitation is that YouTube does not allow the same search control as bibliographic databases, such as combining synonymous terms with Boolean operators. Therefore, some videos using colloquial terms may not have been retrieved, even though the search identified titles containing expressions such as “El Chagas” and “Mal de Chagas”. Finally, the present study emphasizes the urgent need for evidence-based, accessible, and engaging educational content on Chagas disease. Health international organizations and professionals should prioritize creating and disseminating high-quality videos that align with clinical guidelines and are tailored to the lay audience.

## Conclusion

There is a significant lack of compliance between YouTube videos on Chagas disease and current scientific guidelines. Health professionals, international organizations, government agencies, and academic institutions should enhance their communication strategies on the platform to address this gap. This includes creating videos that accurately cover key topics of the disease, such as its definition, etiology, natural history, diagnosis, and treatment, while using accessible language and keeping the content concise to ensure broader public engagement and understanding.

## Supplementary Information

Supplementary Information.

## Data Availability

Data are available upon reasonable request. The datasets generated and analyzed during the current study are available through contact with the corresponding authors: whesleytanor@gmai.com.
