# Supplementary material for: Quality of YouTube videos on Chagas disease: compliance with clinical guidelines
Source: Sci Rep. 2026 May 1;16:20237. doi: 10.1038/s41598-026-50600-4 (PMC13324721; doi:10.1038/s41598-026-50600-4)
Supplement: Supplementary file 1 — Supplementary Information. [file 41598_2026_50600_MOESM1_ESM.docx]

**Appendix 1: Term of commitment for data use**

*Title of research project:* Analysis of quality of informative content on Chagas disease presented in YouTube videos.

The coordinators researchers of this research project undertake to preserve the identity of the authors of the videos under study available on the YouTube website. The data will be used exclusively for this research carried out at the Universidade Federal dos Vales do Jequitinhonha e Mucuri and Oswaldo Cruz Foundation (Fiocruz). The researchers agree that this information will be used solely and exclusively for scientific purposes. Information may only be disclosed anonymously.


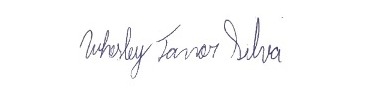


**Whesley Tanor Silva**

Department of Physiotherapy, Federal University of Jequitinhonha and Mucuri Valleys

# Evandro Chagas National Institute of Infectious Diseases

# Oswaldo Cruz Foundation (Fiocruz)

# Corresponding author on behalf of co-authors

**Appendix 2: First version of the scale for assessing content compliance with guidelines**

***Definition***

Chagas disease is a condition caused by the parasite T. Cruzi, discovered by the Brazilian scientist Carlos Chagas in 1909. It is an endemic condition in Latin America and has started to happen in non-endemic countries.

() adequate;

() not adequate;

***Etiology***

The etiological agent is Trypanosoma Cruzi, which can be contracted by vector transmission (by the barber sting), transfusional transmission, congenital transmission, laboratory accidents, oral transmission and/or transmission in organ transplants.

0 (); 1 (); 2 (); 3 (); 4 (); 5 (); 6 ();

***Natural history of the disease***

Decades after infection, approximately 60% of infected individuals remain free of clinical manifestations of the disease for life, 10% develop gastrointestinal disease and 30% a 40% develop Chagas cardiomyopathy and a part can develop the mixed form (both simultaneous attacks).

0 (); 1(); 2 (); 3 (); 4 ()

***Pathogenic hypotheses***

The onset and progression of chagas disease include: 1) direct tissue damage induced by parasites; 2) indirect inflammatory/immunological tissue damage; 3) neurogenic disorders; 4) microvascular disorders.

0 (); 1 (); 2 (); 3 (); 4 ();

***Diagnosis***

Combination of two positive serological tests (ELISA, indirect hemagglutination [HAI] or indirect immunofluorescence [IIF]) and the eventual application of a third one, if the results of these are discordant, to reach a definitive diagnosis. In addition, compatible electrocardiographic and clinical findings are required.

0 () No information according to guidelines;

1 () Mentioned at least a serological test recognized by the guidelines;

2 () Mentioned the recommendation of at least two tests (three in case of discrepancies)

3 () Mentioned recommendation of at least two tests (three, in case of discrepancies) plus clinical and electrocardiographic evaluation;

***Treatment***

Antiparasitic treatment

There are only two drugs used in antiparasitic treatment, which are Nifurtimox (nitrofuran) and Benznidazole (nitroimidazole). In Brazil, only Benznidazole is used.

Antiparasitic treatment

There are only two drugs used in antiparasitic treatment, which are Nifurtimox (nitrofuran) and Benznidazole (nitroimidazole). In Brazil, only Benznidazole is used.

This treatment is indicated if:

- Acute phase for every patient, irrespective of the transmission mechanism;
- Children in the chronic phase;
- Accidental contamination (laboratory);
- In cases of relapse (usually in immunosuppressed patients)
- Patients in the indeterminate chronic phase or with mild clinical presentations (there is no evidence)

0 () No information according to guidelines;

1 () He only mentioned that there is antiparasitic treatment or clinical control of the disease;

2 () Cited indications only for antiparasitic treatment or for clinical control treatments;

3 () He cited the indications for antiparasitic treatment and for clinical control treatments;

**Appendix 3: Final version of the scale**

5-topic scale to verify the quality of information about Chagas disease by matching videos available on YouTube with current guidelines.

| **Topic 1**: Definition of the disease | |
| --- | --- |
| Expected content: “Chagas disease is an infection caused by the parasite *T. cruzi*, discovered by the Brazilian scientist Carlos Chagas in 1909. It is an endemic condition in Latin America; however, prevalence rates are increasing in non-endemic regions.” | |
| Classification: | ( ) adequate |
|  | ( ) not adequate |
| **Topic 2**: Etiology | |
| Expected content: The etiological agent is *Trypanosoma cruzi*, which can be transmitted by triatomines (also known as kissing bugs), blood transfusion, congenital transmission, laboratory accidents, oral transmission and/or organ transplants. | |
| Score: | 1 The video mentioned the etiological agent but did not the means of transmission |
|  | 2 The video mentioned the etiological agent and, at least, two means of transmission |
|  | 3 The video mentioned the etiological agent and more than two means of transmission |
| **Topic 3**: Natural history of the disease | |
| Expected content: Decades after infection, approximately 60% of infected individuals remain asymptomatic or without important clinical changes; 10% develop gastrointestinal abnormalities, 30% a 40% will progress with heart disease, and a part can develop the mixed form (with cardiac and gastrointestinal involvement). | |
| Score: | 1 The video mentioned that there are acute or chronic phases |
|  | 2 The video mentioned that there are acute and chronic phases |
|  | 3 The video mentioned that there are acute and chronic phases and mentioned the chronic forms of the disease |
| **Topic 4**: Diagnosis | |
| Expected content: Combination of two positive serological tests (ELISA, indirect hemagglutination [HAI] or indirect immunofluorescence [IIF]) and the eventual application of a third one, if the results of these are discordant, to reach a definitive diagnosis. In addition, compatible electrocardiographic and clinical findings are required. | |
| Score: | 1 The video mentioned at least a serological test recognized by the guidelines |
|  | 2 The video mentioned the recommendation of at least two tests (three in case of discrepancies) |
|  | 3 The video mentioned recommendation of at least two tests (three, in case of discrepancies) in addition to clinical and electrocardiographic evaluation (to assess possible complications, not to diagnose the disease). |
| **Topic 5**: Treatment | |
| Expected content: There are only two drugs used in antiparasitic treatment: Nifurtimox (nitrofuran) and Benznidazole (nitroimidazole). In Brazil, only Benznidazole is used.  This treatment is indicated if:   - Acute phase for every patient, irrespective of the transmission mechanism; - Children in the chronic phase; - Accidental contamination (laboratory); - In cases of relapse (usually in immunosuppressed patients) - Patients in the indeterminate chronic phase or with mild clinical presentations (there is no evidence) | |
| Score: | 1 The video only mentioned that there is antiparasitic treatment or clinical control of the disease |
|  | 2 The video cited indications only for antiparasitic treatment OR for clinical control treatments |
|  | 3 The video cited the indications for antiparasitic treatment AND for clinical control treatments |

**Table 1. Individual continuous ratings in the pilot sample**

| Video | Definition of the disease (R1) | Definition of the disease (R2) | Etiology (R1) | Etiology (R2) | Natural history of the disease (R1) | Natural history of the disease (R2) | Diagnosis (R1) | Diagnosis (R2) | Treatment (R1) | Treatment (R2) |
| --- | --- | --- | --- | --- | --- | --- | --- | --- | --- | --- |
| 1 | + | + | 3 | 3 | 3 | 3 | 2 | 2 | 3 | 3 |
| 2 | + | + | 3 | 3 | 1 | 1 | 1 | 0 | 1 | 2 |
| 3 | - | - | 2 | 2 | 1 | 1 | 0 | 0 | 0 | 1 |
| 4 | + | + | 2 | 2 | 1 | 2 | 2 | 2 | 2 | 2 |
| 5 | + | + | 2 | 2 | 2 | 2 | 0 | 0 | 0 | 0 |
| 6 | + | + | 2 | 2 | 1 | 0 | 0 | 0 | 0 | 0 |
| 7 | + | + | 3 | 3 | 1 | 0 | 0 | 0 | 3 | 3 |
| 8 | + | - | 2 | 1 | 2 | 2 | 1 | 0 | 3 | 2 |
| 9 | + | + | 2 | 2 | 3 | 3 | 0 | 0 | 0 | 1 |
| 10 | + | + | 3 | 2 | 2 | 1 | 0 | 0 | 1 | 3 |
| 11 | + | + | 3 | 2 | 1 | 0 | 1 | 0 | 2 | 2 |
| 12 | + | + | 2 | 2 | 3 | 3 | 0 | 0 | 0 | 0 |

**Table 2. Inter-rater agreement in the pilot sample after dichotomization of domain scores**

| Domain | n | Agreement (%) | Cohen’s kappa | p-value |
| --- | --- | --- | --- | --- |
| Definition of the disease | 12 | 91.7 | 0.625 | 0.0195 |
| Etiology* | 12 | 91.7 | 0.000 | NA |
| Natural history of the disease | 12 | 83.3 | 0.667 | 0.0209 |
| Diagnosis | 12 | 100.0 | 1.000 | <0.001 |
| Treatment | 12 | 83.3 | 0.676 | 0.013 |

*For the etiology domain, although observed agreement was high, the kappa coefficient was affected by the concentration of ratings in the same category after dichotomization

**Table 3. Characteristics of the included videos (n=96)**

| Variable | | Values |
| --- | --- | --- |
| Views (median, 25-75%IQR) | | 8425.50 (1036.25 – 42499.25) |
| Likes (median, 25-75%IQR) | | 147.50 (17.50 – 862.25) |
| Duration in min (median, 25-75%IQR) | | 4.49 (2.69 – 20.33) |
| Country of origin (n, %) | Brazil | 47 (49.00%) |
|  | USA | 11 (11.50%) |
|  | Chile | 1 (1.00%) |
|  | Argentina | 7 (8.61%) |
|  | El Salvador | 2 (2.10%) |
|  | Spain | 9 (9.40%) |
|  | Peru | 1 (1.00%) |
|  | Switzerland | 4 (4.50%) |
|  | Colombia | 1 (1.00%) |
|  | Canada | 2 (2.10%) |
|  | UK | 1 (1.00%) |
|  | Mexico | 1 (1.00%) |
|  | Australia | 1 (1.00%) |
|  | Denmark | 1 (1.00%) |
|  |  |  |
| Upload source (n, %) | Healthcare and academic institutions/professionals | 27 (28.1%) |
|  | Governmental/intergovernmental organizations | 29 (30.2%) |
|  | Others | 40 (41.7%) |
| Language |  |  |
|  | Portuguese | 46 (47.9%) |
|  | Spanish | 32 (33.3%) |
|  | English | 18 (18.8%) |

Data are shown as median and 25-75% interquartile range or absolute frequency and percentage. USA: United States of America; UK: United Kingdom.

**Table 4. Univariate analysis for the association between video characteristics and quality.**

**Table 4.1. Definition**

| Characteristic | PR | 95% CI | p-value |
| --- | --- | --- | --- |
| Views (per 1000 unit) | 0.996 | 0.992 to 1.000 | 0.06 |
| Likes (per 100 unit) | 1.002 | 0.989 to 1.016 | 0.72 |
| Duration (per min) | 0.998 | 0.996 to 0.999 | **0.04** |
| Country of Origin |  |  |  |
| Non-endemic region | Reference | | |
|  |  |  |  |
| Endemic region | 1.51 | 0.89 to 2.56 | 0.12 |
| Language | – | – |  |
| Portuguese | Reference | | |
|  |  |  |  |
| English | 0.76 | 0.46 to 1.27 | 0.31 |
| Spanish | 0.33 | 0.16 to 0.67 | **0.02** |
| Upload source |  |  |  |
| Others | Reference | | |
| Governmental/intergovernmental organizations | 1.37 | 0.81 to 2.35 | 0.23 |
| Healthcare and academic institutions/professionals | 1.58 | 0.95 to 2.62 | 0.07 |

**Table 4.2. Etiology**

| Characteristic | PR | 95% CI | p-value |
| --- | --- | --- | --- |
| Views (per 1000 unit) | 1.001 | 1.000 to 1.002 | **0.006** |
| Likes (per 100 unit) | 1.005 | 1.001 to 1.007 | **0.003** |
| Duration (per min) | 1.000 | 1.000 to 1.001 | **<0.001** |
| Country of origin |  |  |  |
| Non-endemic country | Reference | – | – |
| Endemic country | 1.06 | 0.85 to 1.31 | 0.57 |
| Language | – | – |  |
| Portuguese | Reference | – | – |
| English | 1.04 | 0.81 to 1.34 | 0.75 |
| Spanish | 0.08 | 0.89 to 1.32 | 0.43 |
| Upload source |  |  |  |
| Others | Reference | – | – |
| Governmental/intergovernmental organizations | 0.95 | 0.73 to 1.21 | 0.67 |
| Healthcare and academic institutions/professionals | 1.08 | 0.89 to 1.32 | 0.42 |

**Table 4.3. Natural history of disease**

| Characteristic | PR | 95% CI | p-value |
| --- | --- | --- | --- |
| Views (per 1000 unit) | 1.001 | 0.999 to 1.002 | 0.13 |
| Likes (per 100 unit) | 1.001 | 0.994 to 1.009 | 0.73 |
| Duration (per min) | 1.000 | 1.000 to 1.001 | **0.001** |
| Country of origin |  |  |  |
| Non-endemic country | Reference | – | – |
| Endemic country | 1.01 | 0.74 to 1.35 | 0.96 |
| Language | – | – |  |
| Portuguese | Reference | – | – |
| English | 1.02 | 0.70 to 1.49 | 0.91 |
| Spanish | 0.98 | 0.72 to 1.35 | 0.94 |
| Upload source |  |  |  |
| Others | Reference | – | – |
| Governmental/intergovernmental organizations | 0.88 | 0.61 to 1.29 | 0.52 |
| Healthcare and academic institutions/professionals | 1.04 | 0.77 to 1.41 | 0.79 |

**Table 4.4. Diagnosis**

| Characteristic | PR | 95% CI | p-value |
| --- | --- | --- | --- |
| Views (per unit) | 0.999999 | 0.999997 to 1.000002 | 0.57 |
| Views (per 1000 unit) | 0.998 | 0.992 to 1.005 | 0.81 |
| Likes (per 100 unit) | 0.997 | 0.970 to 1.024 | 0.81 |
| Duration (per min) | 0.998 | 0.996 to 0.999 | **0.03** |
| Endemic country | 4.05 | 0.60 to 27.35 | 0.96 |
| Non-endemic country | Reference | – | – |
| Language | – | – |  |
| Portuguese | Reference | – | – |
| English | NE | NE | NE |
| Spanish | 1.18 | 0.46 to 3.03 | 0.72 |
| Upload source |  |  |  |
| Others | Reference | – | – |
| Governmental/intergovernmental organizations | 1.50 | 0.33 to 6.70 | 0.59 |
| Healthcare and academic institutions/professionals | 1.53 | 0.38 to 6.23 | 0.54 |

NE: Not estimated due to complete separation.

**Table 4.5. Treatment**

| Characteristic | PR | 95% CI | p-value |
| --- | --- | --- | --- |
| Views (per unit) | 0.994 | 0.987 to 1.000 | 0.07 |
| Likes (per unit) | 0.985 | 0.963 to 1.007 | 0.18 |
| Duration (per min) | 1.001 | 1.000 to 1.001 | **<0.001** |
| Country of origin |  |  |  |
| Non-endemic country | Reference | – | – |
| Endemic country | 0.71 | 0.42 to 1.16 | 0.17 |
| Language | – | – |  |
| Portuguese | Reference | – | – |
| English | 2.12 | 1.18 to 3.81 | **0.01** |
| Spanish | 1.42 | 0.77 to 2.62 | 0.26 |
| Upload source |  |  |  |
| Others | Reference | – | – |
| Governmental/intergovernmental organizations | 1.53 | 0.64 to 3.70 | 0.34 |
| Healthcare and academic institutions/professionals | 3.22 | 1.57 to 6.59 | **0.001** |

NE= Not estimated (complete separation problem); Healthcare/Academic = Healthcare/academic institutions.

**Table 5. Complete multivariable models for adequate quality according to content topic, including variables entered before final model reduction**

| Topic | Variable | Category | PR | 95% CI | p-value |
| --- | --- | --- | --- | --- | --- |
| Definition |  |  |  | | |
|  | Upload source | Others | Reference | | |
|  |  | Governmental/intergovernmental organizations | 1.47 | 0.92 to 2.35 | 0.11 |
|  |  | Healthcare and academic institutions/professionals | 1.86 | 1.14 to 3.02 | **0.01** |
|  | Language | Portuguese | Reference | | |
|  |  | English | 0.57 | 0.34 to 0.97 | **0.03** |
|  |  | Spanish | 0.31 | 0.16 to 0.61 | **0.01** |
|  | Views |  | 0.997 | 0.99 to 1.00 | 0.14 |
|  | Duration (per 5 min) |  | 0.998 | 0.995 to 0.999 | **0.02** |
| Etiology |  |  |  |  |  |
|  | Views  (per 1000 units) |  | 1.000 | 1.000 to 1.001 | **0.02** |
|  | Likes  (per 100 units) |  | 1.003 | 1.001 to 1.006 | **0.03** |
|  | Duration (per 5 min) |  | 1.000 | 1.000 to 1.001 | **<0.001** |
| Natural history of disease |  |  |  |  |  |
|  | Duration (per 5 min) |  | 1.000 | 1.000 to 1.001 | **0.001** |
| Diagnosis |  |  |  |  |  |
|  | Duration (per 5 min) |  | 0.998 | 0.996 to 0.999 | **0.03** |
| Treatment |  |  |  |  |  |
|  | Upload source | Others | Reference | | |
|  |  | Governmental/intergovernmental organizations | 1.75 | 0.68 to 4.47 | 0.24 |
|  |  | Healthcare and academic institutions/professionals | 3.69 | 1.67 to 8.15 | **0.001** |
|  | Language | Portuguese |  | Reference |  |
|  |  | English | 1.44 | 0.77 to 2.71 | 0.25 |
|  |  | Spanish | 1.34 | 0.76 to 2.34 | 0.31 |
|  | Views |  | 0.997 | 0.991 to 1.003 | 0.36 |
|  | Duration (per 5 min) |  | 1.001 | 1.001 to 1.002 | **<0.001** |

PR for Views is expressed per 1000 views; for Likes per 100 likes; for Duration per 5 minutes. Abbreviations: CI, confidence interval; PR, prevalence ratio; ref., reference category; NHD, natural history of disease.
